# Serum Bilirubin Affects Graft Outcomes through UDP-Glucuronosyltransferase Sequence Variation in Kidney Transplantation

**DOI:** 10.1371/journal.pone.0093633

**Published:** 2014-04-01

**Authors:** Jung Pyo Lee, Do Hyoung Kim, Seung Hee Yang, Jin Ho Hwang, Jung Nam An, Sang Il Min, Jongwon Ha, Yun Kyu Oh, Yon Su Kim, Chun Soo Lim

**Affiliations:** 1 Department of Internal Medicine, Seoul National University Boramae Medical Center, Seoul, Korea; 2 Department of Internal Medicine, Chung-Ang University Hospital, Seoul, Korea; 3 Department of Internal Medicine, Seoul National University College of Medicine, Seoul, Korea; 4 Kidney Research Institute, Seoul National University College of Medicine, Seoul, Korea; 5 Department of Surgery, Seoul National University College of Medicine, Seoul, Korea; University of Florence, Italy

## Abstract

**Background:**

Oxidative stress is a major mediator of adverse outcome after kidney transplantation. Bilirubin is produced by heme oxygenase-1 (HO-1), catalyzed by UDP-glucuronosyltransferase (UGT1A1), and has potential as an antioxidant. In this study, we investigated the effects of HO-1 and UGT1A1 sequence variations on kidney allograft outcomes.

**Methods:**

Clinical data were collected from 429 Korean recipients who underwent kidney transplantation from 1990–2008. Genotyping for UGT1A1*28 and HO-1 (A−413T) was performed. Acute rejection and graft survival were monitored as end-points.

**Results:**

Serum levels of total bilirubin were significantly increased after transplantation (0.41±0.19 mg/dL to 0.80±0.33 mg/dL, *P*<0.001). Post-transplant 1-year bilirubin level was higher in 6/7 or 7/7 carriers compared with 6/6 homozygotes in terms of the UGT1A1*28 polymorphism (6/6 vs. 6/7 vs. 7/7: 0.71±0.27 vs. 1.06±0.36 vs. 1.10±0.45 mg/dL, *P*<0.001). According to an additive model of genotype analysis, the 7-allele genotype had a protective effect on the development of acute rejection compared with the 6-allele (odds ratio 0.43, 95% CI 0.25–0.73, *P* for trend = 0.006). Multivariate Cox regression analysis revealed that individuals carrying the 7-allele had a decreased risk of graft loss, by a factor of 0.36 (95% CI 0.15–0.85, *P* = 0.019). The HO-1 (A−413T) polymorphism had no effect on serum bilirubin levels or graft outcomes.

**Conclusions:**

The UGT1A1*28 polymorphism is associated with changes in serum bilirubin and with graft outcome after kidney transplantation.

## Introduction

Kidney transplantation can restore an accelerated oxidative stress in chronic renal failure to near normal [1]. However, oxidative stress is increased by various causes after transplantation such as ischemia-reperfusion injury, allo-immune activation, and infection. Enhanced oxidative stress affects the development of acute rejection or longterm outcome [2], [3]. Therefore, suppression of oxidative stress is able to improve the longterm survival of transplanted organ [4].

Heme oxygenase-1 (HO-1) plays a role in attenuating the production of bilirubin through its ability to degrade heme [5], [6]. Meanwhile, the activity of hepatic bilirubin uridine diphosphate-glucuronosyltransferase (UGT1A1) regulates the degradation of serum bilirubin [7]. Bilirubin is not only an end product of heme degradation, but is also a potent antioxidant [8], [9], which scavenges reactive oxygen species (ROS) *in vitro*, reduces oxidant-mediated cellular damage, and attenuates oxidant stress *in vivo* [10]. Bilirubin improves renal vascular resistance, urine output, glomerular filtration rates, tubular function, and mitochondrial integrity after ischemia-reperfusion injury [11].

We recently reported that mildly elevated serum bilirubin levels could reduce the risk of end-stage renal disease [12], and an HO-1 polymorphism was found to be a risk factor for mortality in patients with IgA nephropathy [13]. In addition, the UGT1A1*28 TA-repeat polymorphism in the gene promoter region was shown to be a significant risk factor for cardiovascular events and mortality in chronic hemodialysis patients [14]. The UGT1A1*28 TA-repeat polymorphism is characterized by the presence of an additional TA repeat in the TATA sequence of the promoter, giving rise to 7, instead of 6 repeats.

However, the roles of the genetic variants and serum bilirubin in the transplantation field have not been thoroughly investigated. The purpose of this study was to investigate the alteration of bilirubin level by genetic variations of HO-1 (A-413T) and UGT1A1*28 in kidney transplantation patients. In addition, the effects of bilirubin level and the genetic variants on kidney allograft outcomes were examined.

## Results

### Baseline characteristics and genetic variations

The demographic information is summarized in Table 1. A total of 429 recipients (281 men (65.5%), mean age (± SD) 34.6±15.5 years) were included. The UGT1A1*28 genotype frequencies for 6/6, 6/7, and 7/7 were 74.4, 23.3, and 2.4%, respectively, and the HO-1 (A−413T) AA, AT, and TT genotype frequencies were 19.8, 53.0, and 27.2%, respectively. The 7-allele frequency of UGT1A1*28 was 14.0% and the A- allele frequency of HO-1 (A−413T) was 46.3%. Observed allele frequencies did not differ significantly from expected allele frequencies based on conformity with the Hardy-Weinberg equilibrium. There were no significant differences in baseline characteristics of patients according to the respective polymorphisms, except in terms of hypertension.

**Table 1:** Baseline characteristics.

	Total	UGT1A1*28			*P* value	HO-1 (A-413T)			*P* value
		6/6	6/7	7/7		AA	AT	TT	
Number	429	319 (74.4)	100 (23.3)	10 (2.4)		78 (19.8)	209 (53.0)	107 (27.2)	
Recipient gender (men (%))	281 (65.5)	213 (66.8)	63 (63.0)	5 (50.0)	0.457	56 (71.8)	136 (65.1)	72 (67.3)	0.558
Recipient age (years)	34.6±15.5	34.7±16.0	33.5±14.2	41.5±7.4	0.283	36.5±15.2	33.8±15.4	34.1±15.7	0.405
Donor gender (men (%))	222 (51.7)	167 (52.4)	49 (49.0)	6 (60.0)	0.733	40 (51.3)	106 (50.7)	56 (52.3)	0.964
Donor age (years)	38.6±11.9	39.2±11.6	37.2±12.7	33.0±9.2	0.119	39.1±11.0	38.4±11.9	39.1±11.6	0.169
No. of HLA mismatches	2.7±1.5	2.7±1.6	2.7±1.4	2.4±1.3	0.744	2.6±1.5	2.7±1.5	2.7±1.5	0.971
Hypertension (%)					0.040				0.002
No	180 (42.0)	138 (43.3)	40 (40.0)	2 (20.0)		30 (38.5)	91 (43.5)	43 (40.2)	
Ex-hypertension	140 (32.6)	94 (29.5)	39 (39.0)	7 (70.0)		14 (17.9)	75 (35.9)	35 (32.7)	
Current hypertension	109 (25.4)	87 (27.3)	21 (21.0)	1 (10.0)		34 (43.6)	43 (20.6)	29 (27.1)	
Diabetes mellitus (%)					0.059				0.226
No	369 (86.0)	275 (86.2)	87 (87.0)	7 (70.0)		66 (84.6)	189 (90.4)	87 (81.3)	
PTDM	30 (7.0)	20 (6.3)	7 (7.0)	3 (30.0)		6 (7.7)	9 (4.3)	10 (6.3)	
Original DM	30 (7.0)	24 (7.5)	6 (6.0)	0 (0.0)		6 (7.7)	11 (5.3)	10 (6.9)	
No. of transplantations (%)					0.281				0.556
1^st^	240 (55.9)	187 (96.4)	49 (98.0)	4 (80.0)		59 (95.2)	105 (96.3)	69 (97.2)	
2^nd^	8 (2.1)	7 (3.6)	1 (2.0)	1 (20.0)		3 (4.8)	4 (3.7)	2 (2.8)	
Donor type (%)					0.750				0.420
Living related	280 (65.2)	211 (66.4)	63 (63.0)	5 (50.0)		54 (70.1)	138 (66.0)	71 (66.4)	
Living unrelated	73 (17.0)	53 (16.7)	17 (17.0)	3 (30.0)		15 (19.5)	32 (15.3)	21 (19.6)	
Deceased	76 (17.7)	54 (17.0)	20 (20.0)	2 (20.0)		8 (10.4)	39 (19.6)	15 (14.0)	
Calcineurin inhibitor (%)					0.050				0.839
Cyclosporin	239 (55.7)	186 (58.3)	49 (49.0)	4 (40.0)		48 (61.5)	114 (54.5)	62 (57.9)	
Tacrolimus	178 (41.5)	128 (40.1)	45 (45.0)	5 (50.0)		28 (35.9)	89 (42.6)	43 (40.2)	
Other	12 (2.8)	5 (1.6)	6 (6.0)	1 (10.0)		2 (2.6)	6 (2.9)	2 (1.9)	
Antimetabolites (%)					0.409				0.053
No	100 (23.3)	78 (24.5)	19 (19.0)	3 (30.0)		20 (25.6)	52 (24.9)	22 (20.6)	
Mycophenolate	233 (54.3)	165 (51.7)	61 (61.0)	7 (70.0)		40 (51.3)	115 (55.0)	53 (49.5)	
Azathioprine	94 (21.9)	74 (23.2)	20 (20.0)	0 (0.0)		16 (20.5)	42 (20.1)	32 (29.9)	
Other	2 (0.5)	2 (0.6)	0 (0.0)	0 (0.0)		2 (2.6)	0 (0.0)	0 (0.0)	
IL-2 receptor blocker (%)	136 (31.7)	96 (30.1)	35 (35)	5 (50)	0.297	24 (30.8)	70 (33.5)	25 (23.4)	0.177
Pre-transplant bilirubin (mg/dL)	0.42±0.19	0.41±0.18	0.44±0.19	0.46±0.33	0.340	0.41±0.16	0.42±0.20	0.41±0.20	0.909
Pre-transplant hemoglobin (g/dL)	10.4±1.9	10.4±2.0	10.4±1.6	9.9±2.5	0.774	9.8±1.8	10.6±2.0	10.2±1.8	0.043
Pre-transplant protein (g/dL)	6.86±0.80	6.88±0.80	6.78±0.83	6.90±0.53	0.756	6.77±0.56	6.94±0.84	6.78±0.83	0.385
Pre-transplant albumin (g/dL)	3.95±0.56	3.97±0.58	3.94±0.51	3.81±0.28	0.724	3.93±0.50	3.97±0.57	3.94±0.57	0.870

DM, diabetes mellitus; PTDM, post-transplant DM. Measured by independent *t*-test for continuous variable and χ^2^ or Fisher's exact test for categorical variables.

### Change in serum bilirubin level after kidney transplantation according to sequence variation

**Figure 1:**
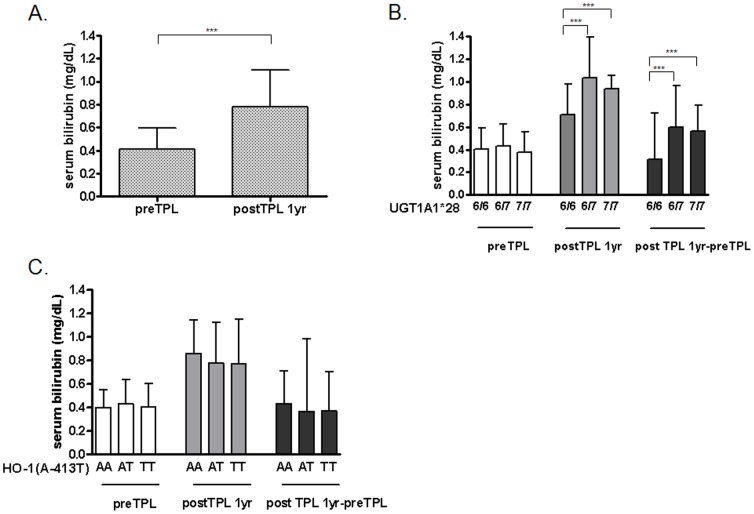
Changes in serum bilirubin levels after kidney transplantation according to sequence variation. (A) Pre- and post-transplant serum bilirubin levels. (B) Serum bilirubin level according to UGT1A1*28 polymorphism. (C) Serum bilirubin level according to HO-1 (A-413T) polymorphism. Mean ± SD. ***P<0.001.

Serum levels of total bilirubin were significantly increased after kidney transplantation (bilirubin: 0.41±0.19 mg/dL to 0.80±0.33 mg/dL, *P*<0.001) (Figure 1A). Pre-transplant serum bilirubin levels were unrelated to UGT1A1*28 polymorphisms. However, UGT1A1*28 polymorphism was significantly associated with serum bilirubin after kidney transplantation. Post-transplant 1-year bilirubin levels were higher in 6/7 or 7/7 carriers compared with 6/6 homozygotes (6/6 vs. 6/7 vs. 7/7: 0.71±0.27 vs. 1.06±0.36 vs. 1.10±0.45 mg/dL, *P*<0.001) (Figure 1B). The HO-1 (A-413T) polymorphism had no effect on serum bilirubin levels at the time of transplantation or 1 year post-transplant (Figure 1C).

### Serum bilirubin level and the development of acute rejection

**Figure 2:**
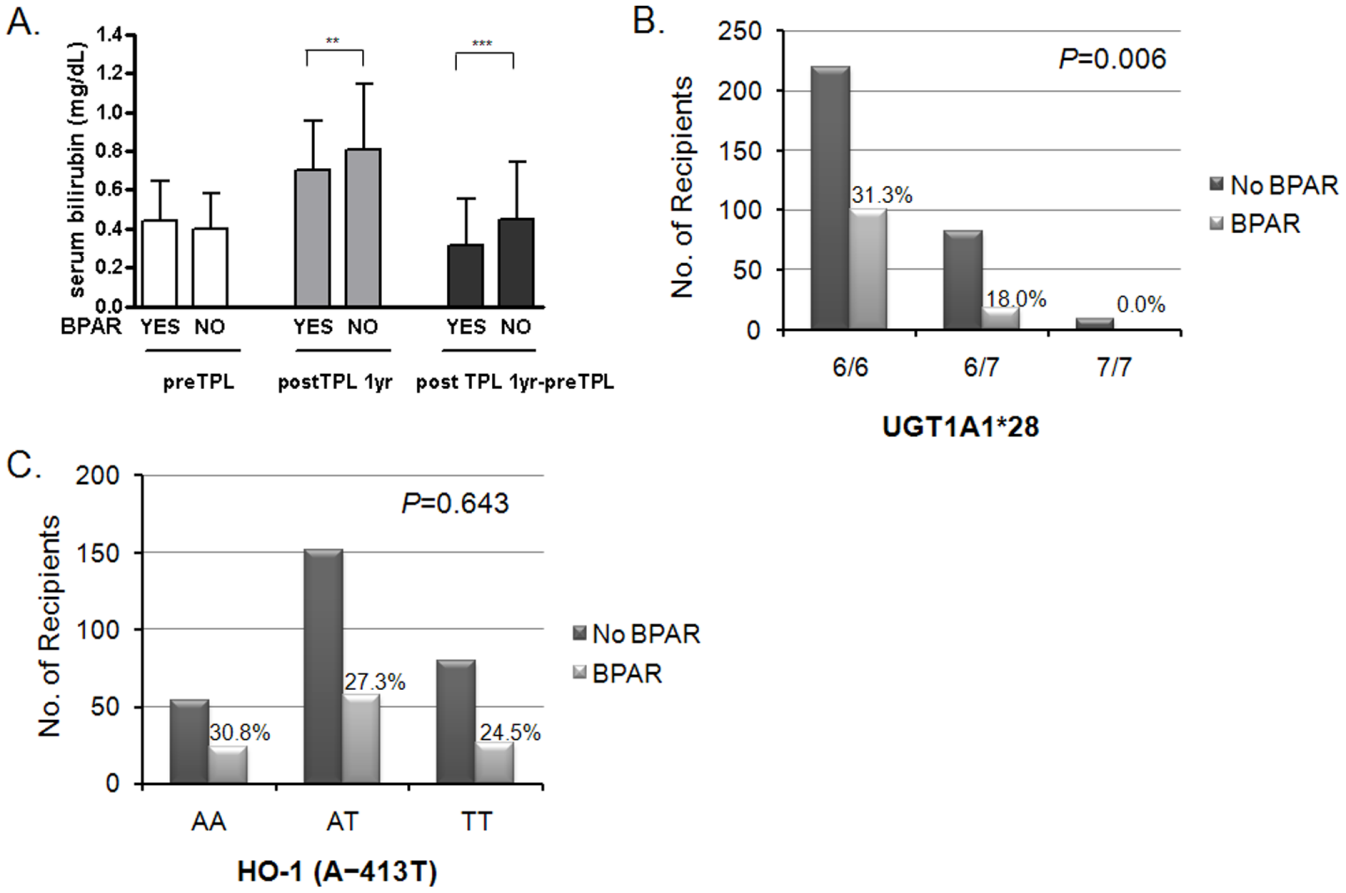
Associations between HO-1 and UGT1A1*28 sequence variations and the development of acute rejection. (A) Pre- and post-transplant serum bilirubin levels according to BPAR. (B) Incidence of BPAR according to UGT1A1*28 polymorphism. (C) Incidence of BPAR according to HO-1 (A-413T) polymorphism. Mean ± SD. TPL, transplantation; postTPL 1yr-preTPL, difference in total bilirubin values between pre-transplant and 1 year post-transplant; BPAR, biopsy-proven acute rejection. **P<0.01; ***P<0.001.

Among the 429 recipients, 118 (27.5%) experienced biopsy-proven acute rejection (BPAR) after renal transplantation. Pre-transplant basal total bilirubin level did not affect the development of acute rejection (AR) (BPAR-positive group vs. negative group 0.44±0.20 vs. 0.40±0.19 mg/dL, *P* = 0.142) (Figure 2A). However, post-transplant 1-year bilirubin levels in the BPAR-negative group were significantly higher than in the BPAR-positive group (0.83±0.35 mg/dL vs. 0.69±0.24 mg/dL, *P* = 0.005). In addition, differences in serum bilirubin between pre-transplantation and 1-year post-transplantation were higher in the BPAR-negative group (BPAR-positive group vs. negative group 0.42±0.34 vs. 0.23±0.31 mg/dL, *P*<0.001).

The 7-allele UGT1A1*28 polymorphism had a protective effect on the development of BPAR compared to the 6-allele (Figure 2B). In the additive model of genotype analysis, the odds ratio of BPAR per copy of donor variant allele (A) was 0.43 (*P* for trend = 0.006, 95% CI 0.25–0.73). Adjustment for multiple covariates did not significantly affect this result (model 3, OR 0.39, 95% CI 0.20–0.76, *P* = 0.006) (Table 2). However, allelic variation of HO-1 (A-413T) was not significantly associated with the occurrence of BPAR (Figure 2C).

**Table 2:** Independent risk of recipients with UGT1A1*28 polymorphism 6/7+7/7 versus 6/6 of acute rejection analyzed by multivariate logistic regression.

	Odds ratio	95% confidence interval	*P* value
HO-1 (A-413T) TT + AT vs. AA			
Model 1^a^	0.81	0.47–1.39	0.436
Model 2^b^	0.76	0.44–1.33	0.340
Model 3^c^	0.88	0.47–1.68	0.707
UGT1A1*28 6/7+7/7 vs. 6/6			
Model 1^a^	0.44	0.25–0.76	0.004
Model 2^b^	0.34	0.19–0.63	0.001
Model 3^c^	0.22	0.09–0.53	0.001

a adjusted for recipient age and recipient gender; ^b^adjusted for recipient age, recipient gender, number of HLA mismatches, donor type; ^c^adjusted for recipient age, recipient gender, number of HLA mismatches, donor type, number of transplantations, type of calcineurin inhibitor, hypertension and diabetes mellitus.

### Effect of serum bilirubin level on long-term graft survival

**Figure 3:**
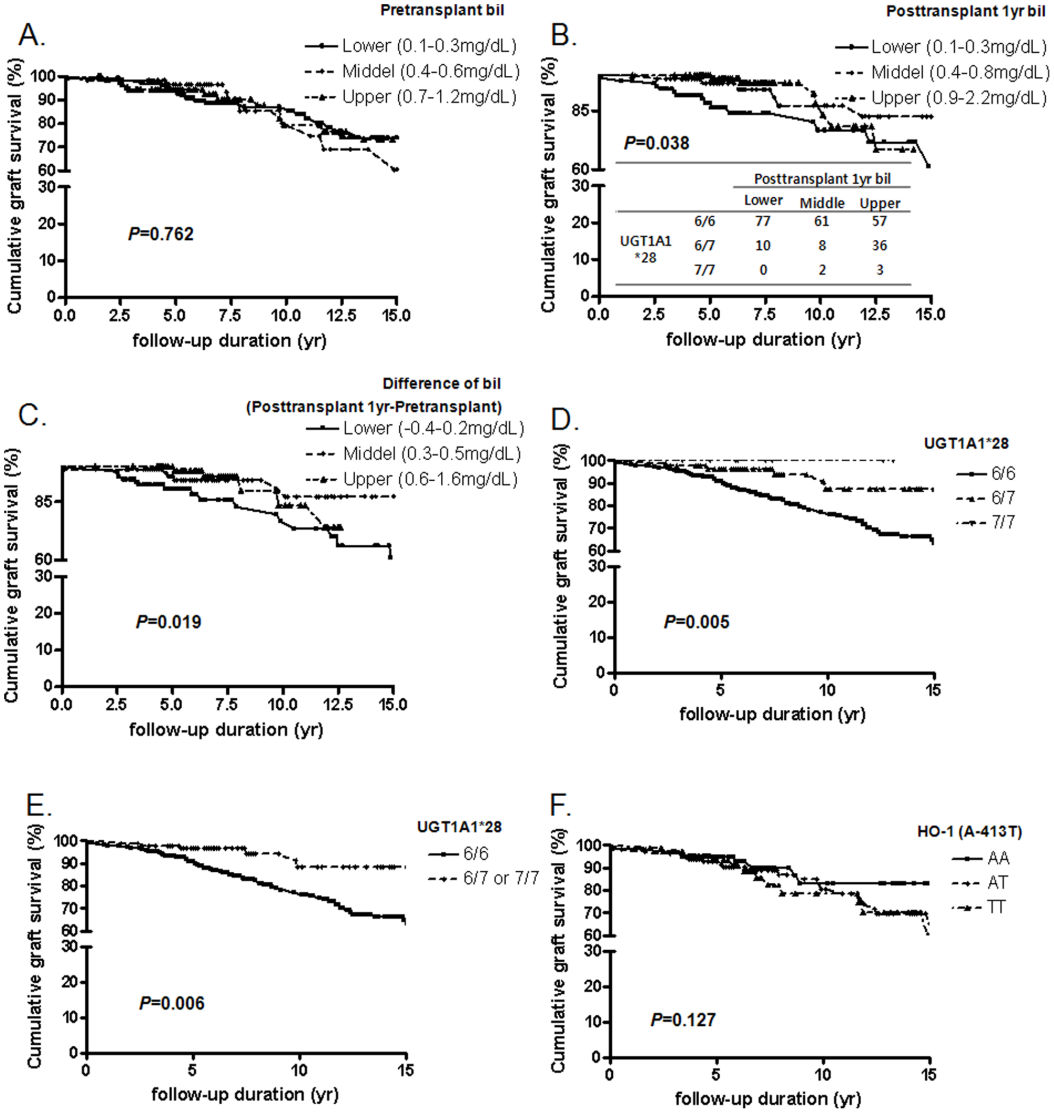
Long-term graft survival and sequence variations of HO-1 or UGT1A1*28. (A) Long-term graft survival according to pre-transplant serum bilirubin levels. (B) Post-transplant serum bilirubin levels. (C) Difference in total bilirubin values between pre-transplant and 1-year post-transplant. (D, E) UGT1A1*28 polymorphisms, and (F) HO-1 (A-413T) polymorphism.

We grouped patients according to the following tertile levels of pretransplant bilirubin, 1-year post-transplant bilirubin, and change of bilirubin. Survival analysis using the Kaplan-Meier method revealed that recipients with higher 1-year bilirubin levels had better graft survival than recipients with lower bilirubin levels (log-rank test for trend *P* = 0.038) (Figure 3B). In addition, the change of serum bilirubin levels between pre-transplantation and 1-year post-transplantation levels showed a similar pattern (log-rank test for trend *P* = 0.019) (Figure 3C). Pre-transplant bilirubin level did not affect graft survival (Figure 3A). The 7-allele UGT1A1*28 polymorphism was a good prognostic factor for graft survival (*P* = 0.005) (Figure 3D, E), but HO-1 (A-413T) polymorphism was not a significant prognostic factor (*P* = 0.762) (Figure 3F).

Unadjusted Cox regression analysis revealed that individuals carrying the 7-allele of UGT1A1*28 polymorphism had a decreased hazard ratio of graft loss (6/7 or 7/7 vs. 6/6 genotype, hazard ratio = 0.53, 95% CI 0.30–0.93, *P* = 0.027). This association was significant after adjusting for several risk factors (Model 4, hazard ratio = 0.36; 95% CI 0.15–0.85, *P* = 0.019) (Table 3).

**Table 3:** Graft-loss risk in recipients with UGT1A1*28 polymorphism 6/7+7/7 versus 6/6 by Cox regression analysis.

	Hazard ratio	95% confidence interval	*P* value
UGT1A1*28 (6/7+7/7 vs. 6/6)			
Model 1^a^	0.53	0.30–0.93	0.027
Model 2^b^	0.54	0.31–0.95	0.034
Model 3^c^	0.52	0.30–0.92	0.025
Model 4^d^	0.36	0.15–0.85	0.019

a unadjusted; ^b^adjusted for recipient age and gender; ^c^adjusted for recipient age, gender, number of HLA mismatches, donor type; ^d^adjusted for recipient age, gender, number of HLA mismatches, donor type, number of transplantations, type of calcineurin inhibitor, hypertension and diabetes mellitus.

## Discussion

This study evaluated the incidence of AR and graft survival following kidney transplantation in relation to genetic polymorphisms in enzymes associated with bilirubin metabolism in a cohort from a single Asian center. After kidney transplantation, serum levels of total bilirubin increased significantly along with the UGT1A1*28 polymorphism, and mildly elevated bilirubin and the 7-allele UGT1A1*28 polymorphism were associated with improved graft outcomes.

Gilbert's syndrome is characterized by mildly elevated unconjugated bilirubin, caused by a genetic variant of the UGT1A1 gene on human chromosome 2. Individuals with Gilbert's syndrome were at lower risk of coronary artery disease [16], [17]. This protective effect was revealed to be associated with an antioxidant effect of bilirubin, rather than with other confounding factors [18]. The UGT1A1*28 polymorphism has recently been shown to be linked to serum bilirubin level and to predict long-term cardiovascular events and mortality in chronic hemodialysis patients [14]. However, to the best of our knowledge, the current study is the first to reveal the effect of serum bilirubin in relation to UGT1A1*28 polymorphism on AR and graft survival after renal transplantation.

Oxidative stress is increased in patients with end-stage renal disease, and results from an imbalance between the production of oxidants and antioxidant defense mechanisms [19]. There have been reports of the effect of oxidative stress on graft function in kidney transplant recipients [20], [21], [22], [23]. Oxidative stress was significantly decreased 1-year post-transplantation, but remained higher than in the healthy control group [21]. In addition, AR was affected by ROS generation during experimental rat kidney transplantation [2].

Bilirubin acts not only as an antioxidant, but has also been shown to have anti-inflammatory properties by anti-complement effects [24]. Because complement is implicated in transplant rejection, bilirubin appears to improve graft outcome through complement-inhibitory activity. Recent studies have revealed that monotherapy with bilirubin [25] or combined therapy with bilirubin, carbon monoxide, and HO-1 [26] can induce immune tolerance by promoting the generation of regulatory T cells. In addition, bilirubin administration led to markedly improved survival of islets transplanted into allogeneic recipients [27]. The results of the present study are in agreement with the results of these earlier studies.

One microsatellite polymorphism in the HO-1 gene promoter has been evaluated in several disease entities, including pulmonary disease [28], [29], cardiovascular disease [30], [31], [32], obstetrics [33], and neurological disease [34]. This microsatellite polymorphism is correlated with susceptibility to coronary artery disease through changing serum bilirubin levels [35]. Our group also documented an association between this polymorphism and the progression of renal disease in IgA nephropathy [13]. This HO-1 gene promoter polymorphism has also been reported to affect the outcomes of renal transplantation by altering HO-1 gene expression [36], [37]. Induction of HO-1 expression appeared to protect islets from apoptosis and rejection in a mouse model of islet transplantation [38], [39]. However, the beneficial effects of this gene on graft outcome remain controversial. Courtney et al. reported no association between this polymorphism and graft or recipient survival in a large cohort [40], and Hribova et al. reported no evidence of an association between renal graft outcomes and the HO-1 (A−413T) promoter polymorphism examined in the current study [41]. Turpeinen et al. also reported that several HO-1 polymorphisms, including these two polymorphisms, had no significant role in renal transplantation outcomes in the Finnish population [42]. The present study found no associations between the HO-1 (A−413T) promoter polymorphism and pre- or post-transplant serum bilirubin levels, AR, or graft survival.

This study had some limitations. First, it was a single-center cohort study, and studies involving larger populations are necessary to confirm our finding that post-transplant changes in serum bilirubin levels affect graft survival. Second, the mechanisms responsible for the post-transplant elevation in bilirubin levels according to the UGT1A1*28 sequence, and the protective effect of high bilirubin levels, need to be investigated at the molecular level.

In conclusion, elevation of serum bilirubin after kidney transplantation was associated with UGT1A1*28 polymorphism. Lower serum bilirubin levels and the 6/6 UGT1A1*28 genotype may have negative effects on graft outcome.

## Methods

### Study population

A total of 429 patients who underwent kidney transplantation at Seoul National University Hospital between 1982 and 2008 were recruited. Recipients with liver diseases, such as carriers of hepatitis B or C virus, were excluded from the study. The research protocol was approved by the Internal Review Board of Seoul National University Hospital. Written informed consent was obtained from all participants and all clinical investigations were conducted according to the principles expressed in the 2000 Declaration of Helsinki.

Medical records of recipients were reviewed based on the electronic medical record system. Clinical parameters that could have influenced graft outcome were collected; i.e., recipient gender and age at transplantation, history of hypertension, presence of diabetes mellitus, cause of end stage renal disease, HLA typing, type of donor, donor-specific antibodies, and use of immunosuppressants. Data on pre-transplant serum protein, albumin, creatinine, and bilirubin levels, as well as post-transplant 1-year serum bilirubin levels were also collected.

### Primary and secondary outcomes

The primary outcome was BPAR. The secondary outcome was graft loss, defined as composite graft dysfunction necessitating new renal replacement therapy after transplantation, or death. Renal replacement therapy data were obtained from the Korean ESRD registry [15]. This registry contains data for patients entering into renal replacement therapy, dialysis or transplantation in Korea from 1985–2012 through an on-line registry program on the KSN website (http://www.ksn.or.kr).

### Determination of genetic polymorphisms

Genomic DNA was extracted from peripheral blood mononuclear cells using a Wizard Genomic DNA Purification Kit (Promega, Madison, WI, USA) according to the manufacturer's instructions.

Genotyping of the UGT1A1*28 TA-repeat polymorphism in the TATA box at position -53 was performed by polymerase chain reaction (PCR) combined with fluorescence technology, as described previously [14]. Briefly, PCR was performed using a 5-carboxyfluorescein (FAM)-labeled forward primer 5′-CACGTGACACAGTCAAAC-3′ and an unlabeled reverse primer 5′-CAACAGTATCTTCCCAGC-3′. Amplification was performed for 34 cycles of denaturation at 94°C for 45 sec, annealing at 62°C for 45 sec, and extension at 72°C for 60 sec between the initial denaturation at 94°C for 2 min and a final extension at 72°C for 1 min. Finally, the PCR products were sequenced to determine the number of TA repeats over the promoter of the UGT1A1*28 gene (Figure S1).

The HO-1 (A−413T) single-nucleotide polymorphism was genotyped using a TaqMan method (7900HT Fast real time PCR system, Applied Biosystems, Foster City, CA, USA). The primer sequences used were 5′-GGGTTGCTAAGTTCCTGATGTTG-3′ (forward) and 5′-CCCAGAAGGTTCCAGAAAGCT-3′ (reverse), and the TaqMan minor groove binder probe sequences were 5′-ACCAGGCTTTTGCTCT-3′ and 5′-ACCAGGCTATTGCTCT-3′. Different fluorescence labels were used to label the 5′ segments of the allelic probes (6-FAM for mutants and 6-carboxy-4,7,2′,7′-tetrachlorofluorescein for wild-type). Reaction mixtures consisted of 1.0 μL 10× AmpliTaq buffer, 1.0 μL deoxynucleotide triphosphates (2.5 mM each), 0.2 μL forward primer (20 pmol/μL), 0.2 μL reverse primer (20 pmol/μL), 1.0 μL genomic DNA (50 ng/μL) and 0.15 μL iMax II Taq polymerase. The PCR reactions were carried out under the following conditions: 5 min at 94°C (one cycle); 30 s at 94°C; 30 s at 56°C (35 cycles); 50 s at 72°C, and 7 min at 72°C (one cycle). PCR products were analyzed on 2% agarose gels. After PCR, the genotype of each sample was attributed automatically by measuring the allele-specific fluorescence using an ABI Prism 7000 Sequence Detection Systems and SDS 1.2.3 software for allele discrimination (Applied Biosystems). Genotypes were confirmed by repeated PCR and DNA sequencing using an ABI Prism BigDye Terminator Kit (Applied Biosystems) in 10% of the study population samples.

### Immunosuppressive treatment protocols

A standardized immunosuppression protocol involving a combination of a calcineurin inhibitor and steroids was initiated within 24 h of surgery. The choice of calcineurin inhibitor (cyclosporine or tacrolimus) was determined by the transplantation team. The initial dose of cyclosporine A was 10 mg/kg/day by the oral route, and target trough levels were 200–400 ng/mL during the first 4 weeks and 100–200 ng/mL thereafter. The initial dose of FK506 was 0.15 mg/kg/day by the oral route, and target trough levels were 8–15 ng/mL during the first 3 months and 3–8 ng/mL thereafter. Methylprednisolone (1 g/day) was administered by intravenous infusion on the day of transplantation, and the dose was then tapered to prednisone 30 mg/day on the fourth day after transplantation. Purine synthesis inhibitors such as mycophenolate mofetil were used as an initial immunosuppressive treatment based on a clinical decision taking into consideration risk factors such as HLA mismatches.

### Statistical analysis

Genotype frequencies were estimated by gene counting. Allele frequencies were deduced from the genotype distribution. Student's *t*-test was used to analyze continuous variables and results are presented as mean ± SD. χ^2^ tests were used to analyze categorical variables. Graft survival was analyzed using the Kaplan-Meier method, and comparisons among groups were performed by log-rank tests. Multivariate analysis was performed using a binary logistic regression test for risk of BPAR and the Cox proportional hazard model for risk of graft loss (backward stepwise method). Variables known to be important risk factors and those that showed a trend towards significance (*P*<0.1) were included in the multivariate models. Because of the significant association between serum bilirubin and HO-1 (A−413T) and UGT1A1*28 polymorphisms, these variables were not analyzed simultaneously in any particular model to avoid multicollinearity. All analyses and calculations were performed using SPSS for Windows package 17.0K (SPSS Inc., Chicago, IL, USA). Values of *P*<0.05 were considered statistically significant.

## Supporting Information

Figure S1 Genotyping of the UGT1A1*28 TA-repeat polymorphism in the TATA box at position -53.
(TIF)
