# Supplementary figures and images for: Serum Bilirubin Affects Graft Outcomes through UDP-Glucuronosyltransferase Sequence Variation in Kidney Transplantation
Source: PLoS One. 2014 Apr 1;9(4):e93633. doi: 10.1371/journal.pone.0093633 (PMC3972238; doi:10.1371/journal.pone.0093633)

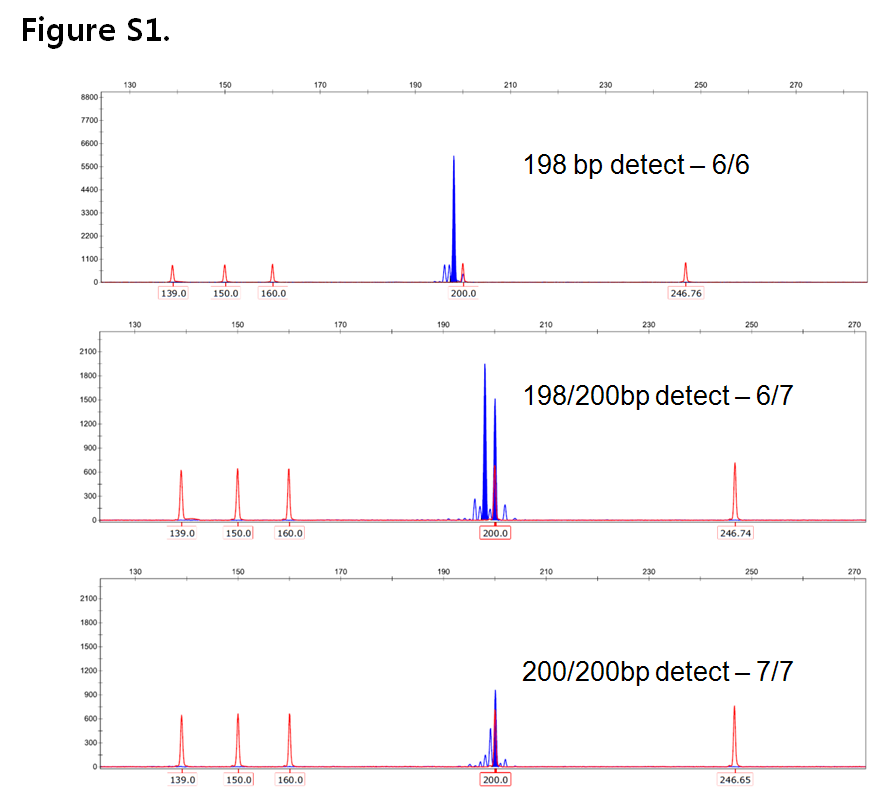

Supplement: Figure S1 — Genotyping of the UGT1A1*28 TA-repeat polymorphism in the TATA box at position -53. (TIF) [file pone.0093633.s001.tif]
